# Supplementary material for: Salt-Specific Suppression of the Cold Denaturation of Thermophilic Multidomain Initiation Factor 2
Source: Int J Mol Sci. 2023 Apr 5;24(7):6787. doi: 10.3390/ijms24076787 (PMC10094840; doi:10.3390/ijms24076787)
Supplement: Supplementary file 1 [file ijms-24-06787-s001.zip › ijms-2229267-supplementary.pptx]

## Slide 1
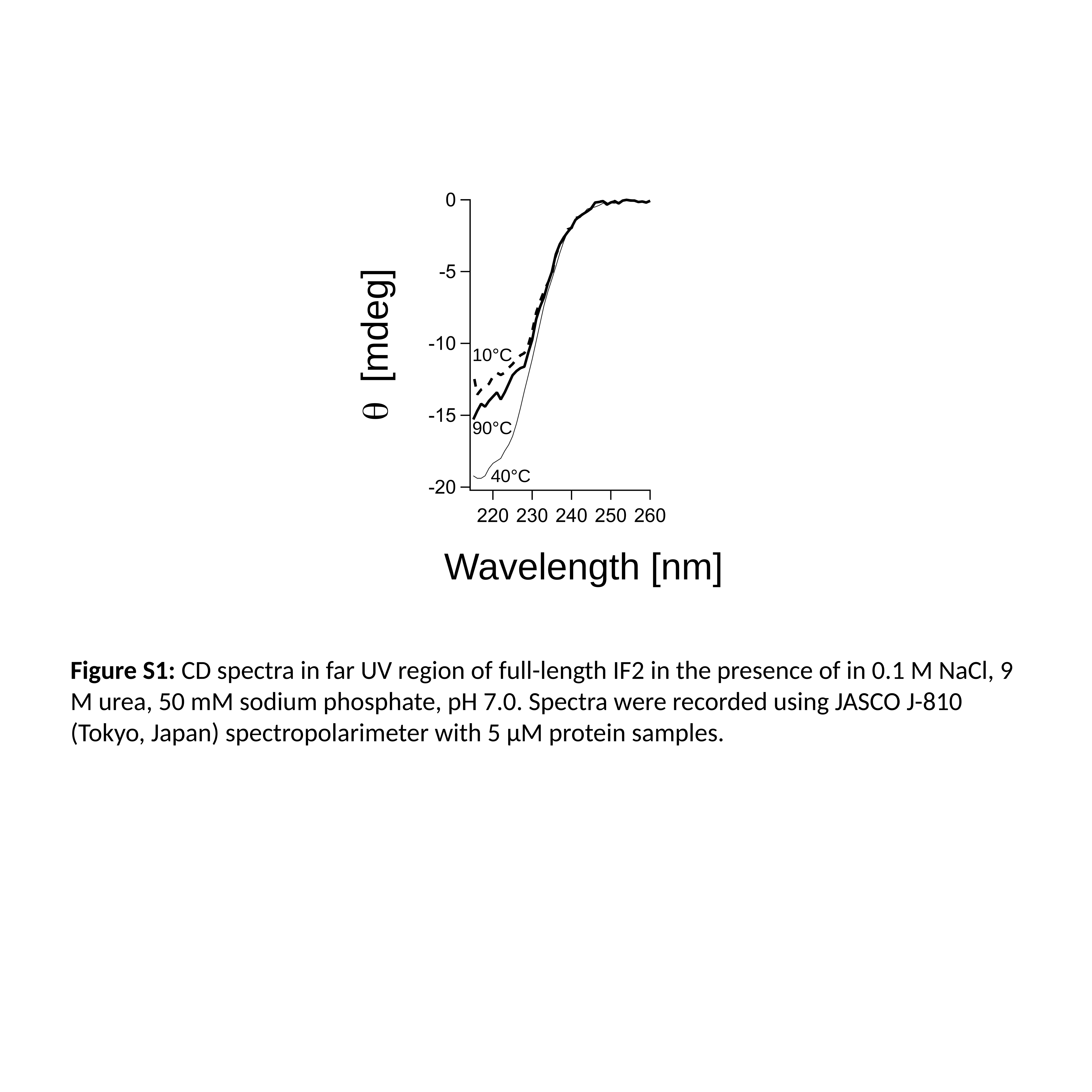

10°C
  [mdeg]
90°C
40°C
Wavelength [nm]
Figure S1: CD spectra in far UV region of full-length IF2 in the presence of in 0.1 M NaCl, 9 M urea, 50 mM sodium phosphate, pH 7.0. Spectra were recorded using JASCO J-810 (Tokyo, Japan) spectropolarimeter with 5 µM protein samples.

## Slide 2
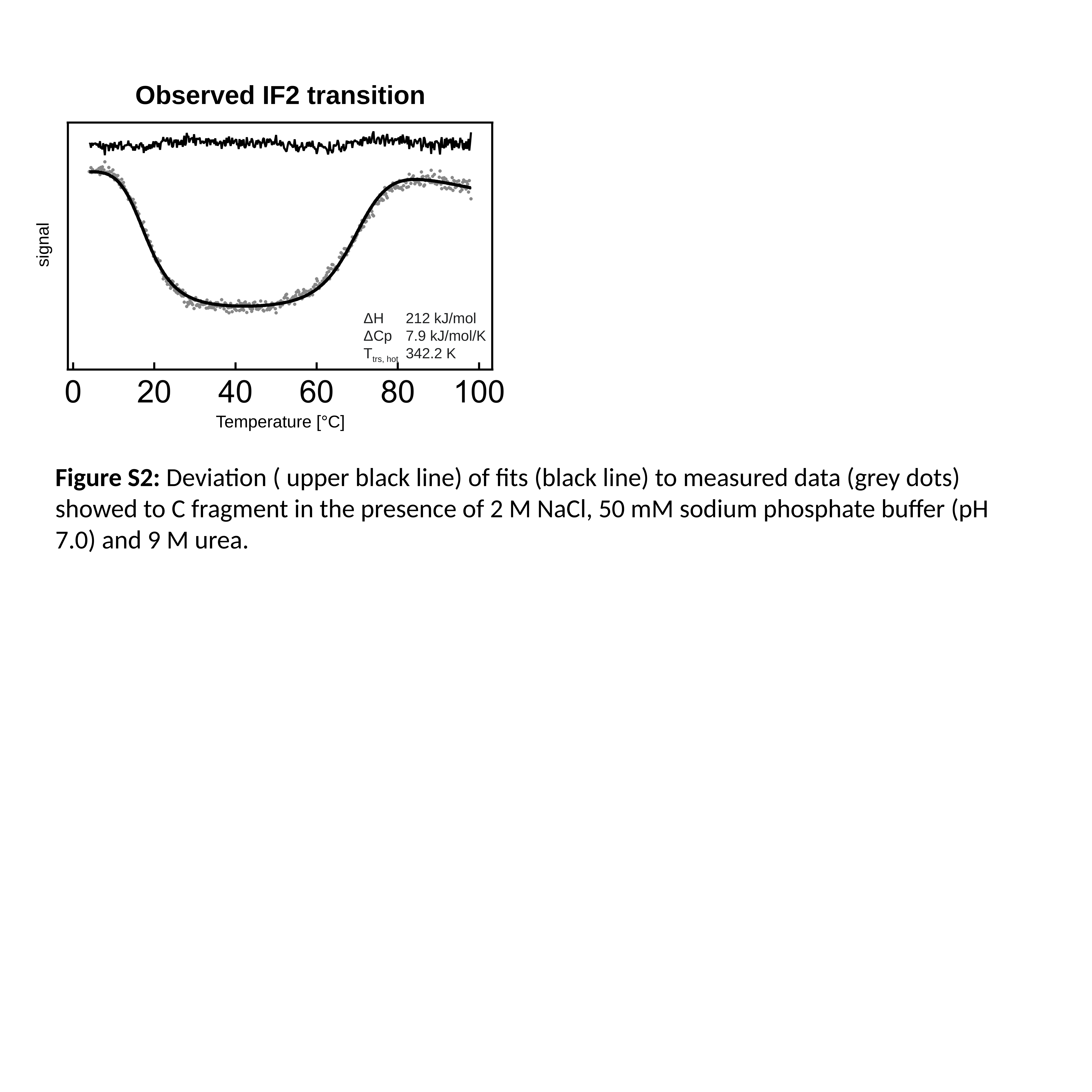

Observed IF2 transition
signal
Temperature [°C]
ΔH
ΔCp
Ttrs, hot
212 kJ/mol
7.9 kJ/mol/K
342.2 K
Figure S2: Deviation ( upper black line) of fits (black line) to measured data (grey dots) showed to C fragment in the presence of 2 M NaCl, 50 mM sodium phosphate buffer (pH 7.0) and 9 M urea.

## Slide 3
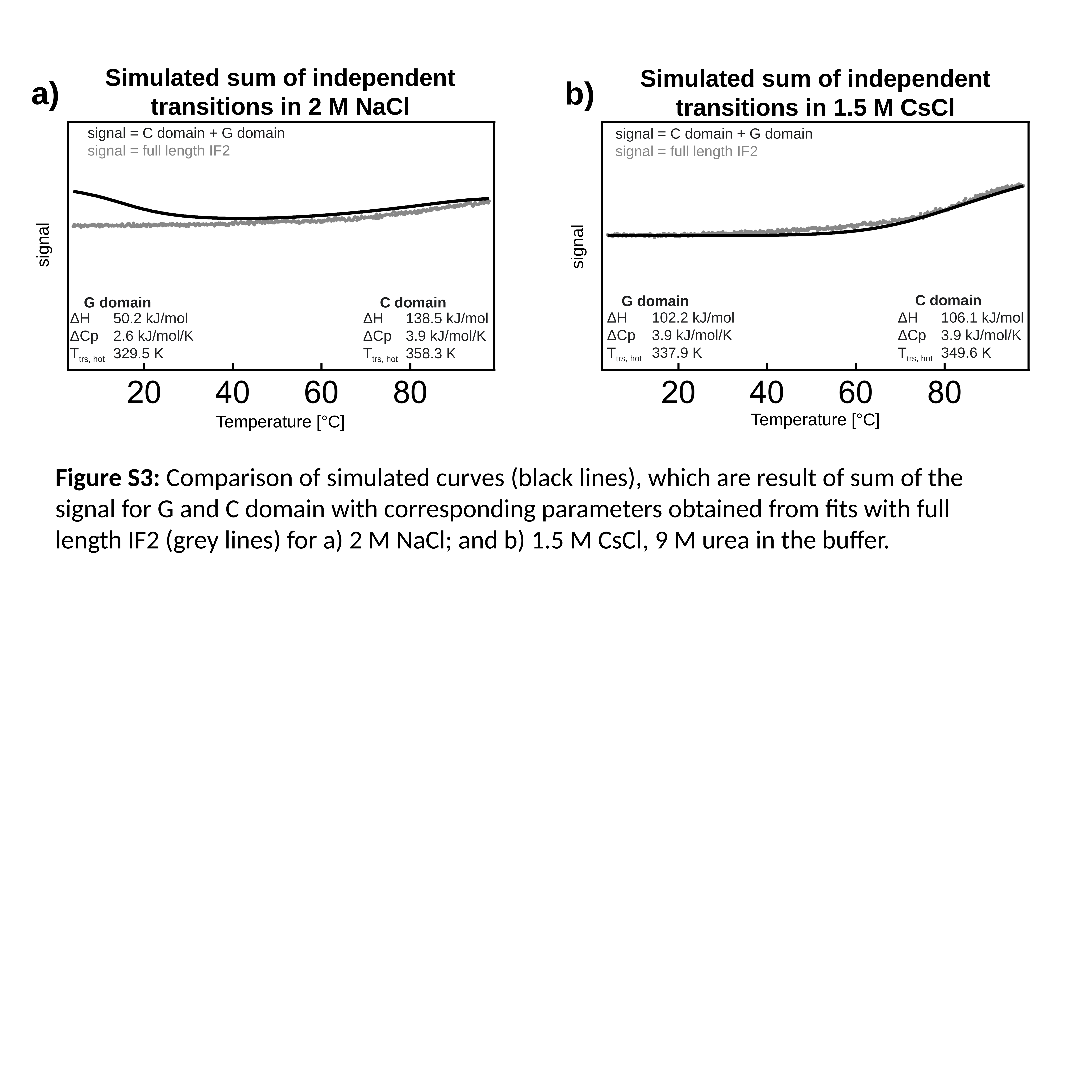

Simulated sum of independent transitions in 2 M NaCl
a)
signal
Temperature [°C]
ΔH
ΔCp
Ttrs, hot
50.2 kJ/mol
2.6 kJ/mol/K
329.5 K
signal = C domain + G domain
signal = full length IF2
G domain
C domain
ΔH
ΔCp
Ttrs, hot
138.5 kJ/mol
3.9 kJ/mol/K
358.3 K
Simulated sum of independent transitions in 1.5 M CsCl
b)
signal
Temperature [°C]
ΔH
ΔCp
Ttrs, hot
102.2 kJ/mol
3.9 kJ/mol/K
337.9 K
signal = C domain + G domain
signal = full length IF2
C domain
G domain
ΔH
ΔCp
Ttrs, hot
106.1 kJ/mol
3.9 kJ/mol/K
349.6 K
Figure S3: Comparison of simulated curves (black lines), which are result of sum of the signal for G and C domain with corresponding parameters obtained from fits with full length IF2 (grey lines) for a) 2 M NaCl; and b) 1.5 M CsCl, 9 M urea in the buffer.

## Slide 4
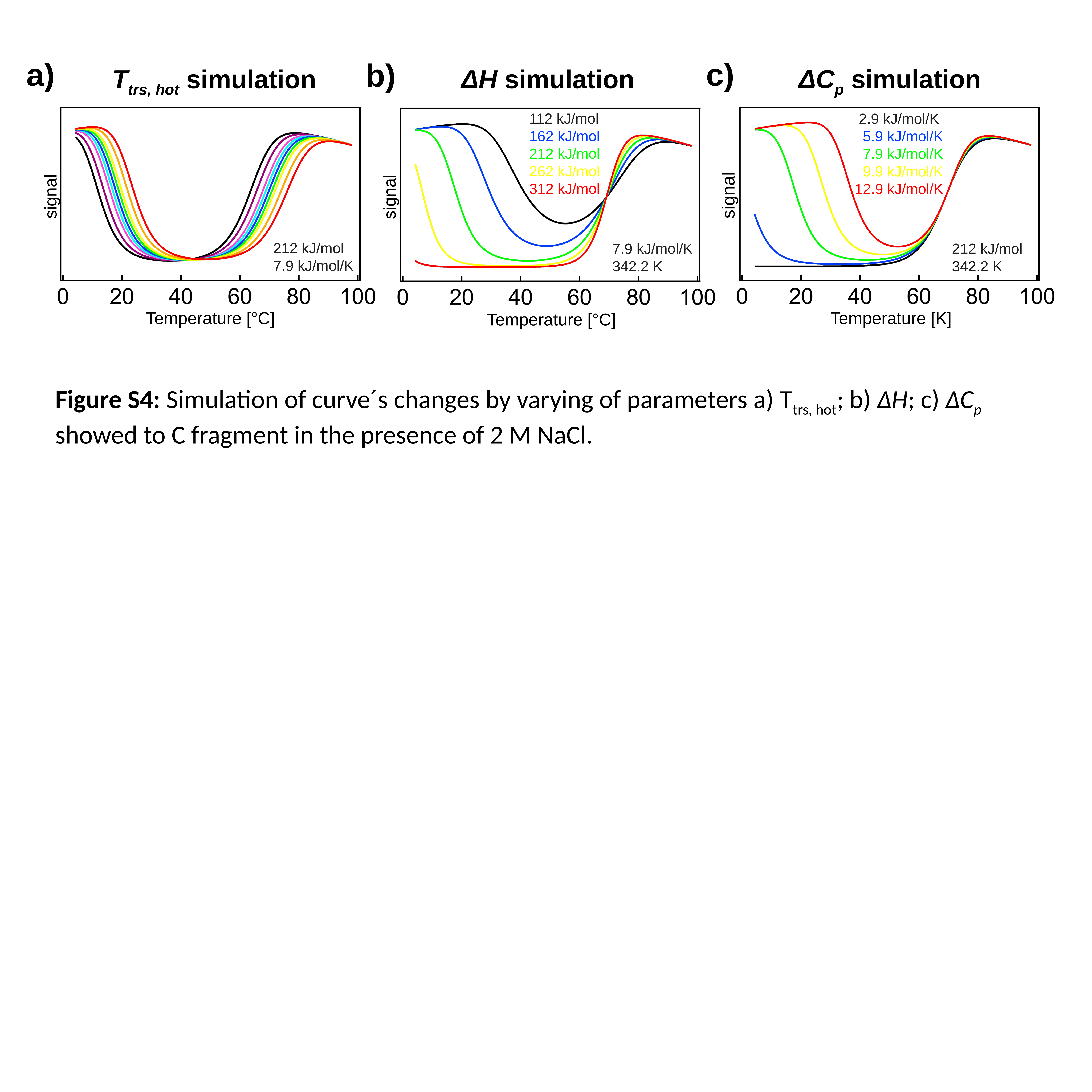

a)
signal
Temperature [°C]
Ttrs, hot simulation
212 kJ/mol
7.9 kJ/mol/K
c)
signal
Temperature [K]
ΔCp simulation
 2.9 kJ/mol/K
 5.9 kJ/mol/K
 7.9 kJ/mol/K
 9.9 kJ/mol/K
12.9 kJ/mol/K
b)
signal
Temperature [°C]
ΔH simulation
112 kJ/mol
162 kJ/mol
212 kJ/mol
262 kJ/mol
312 kJ/mol
7.9 kJ/mol/K
342.2 K
212 kJ/mol
342.2 K
Figure S4: Simulation of curve´s changes by varying of parameters a) Ttrs, hot; b) ΔH; c) ΔCp showed to C fragment in the presence of 2 M NaCl.

## Slide 5
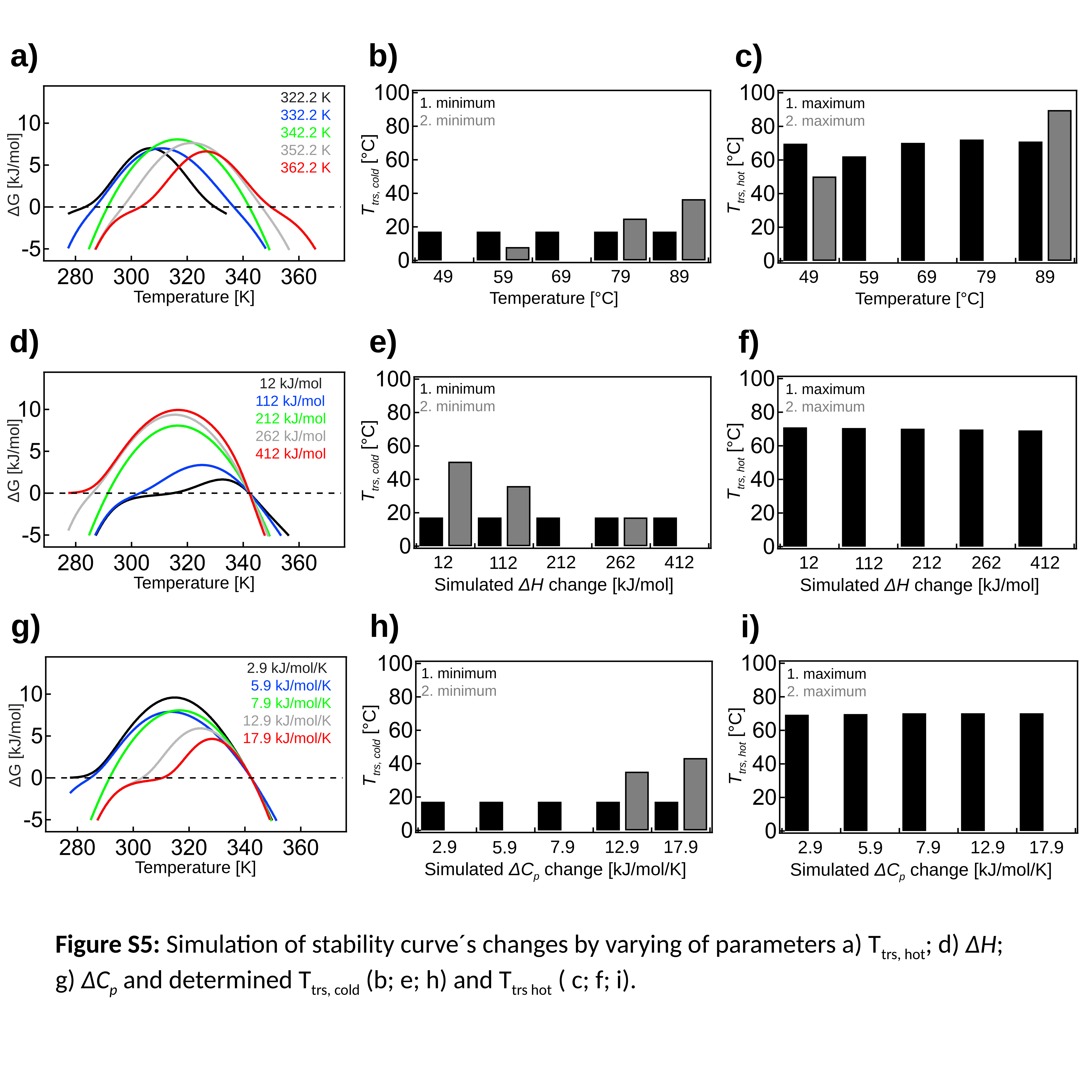

a)
ΔG [kJ/mol]
Temperature [K]
322.2 K
332.2 K
342.2 K
352.2 K
362.2 K
b)
Ttrs, cold [°C]
Temperature [°C]
1. minimum
2. minimum
49
69
79
89
59
c)
Ttrs, hot [°C]
Temperature [°C]
1. maximum
2. maximum
49
69
79
89
59
d)
ΔG [kJ/mol]
Temperature [K]
 12 kJ/mol
112 kJ/mol
212 kJ/mol
262 kJ/mol
412 kJ/mol
e)
Ttrs, cold [°C]
Simulated ΔH change [kJ/mol]
1. minimum
2. minimum
12
212
262
412
112
f)
Ttrs, hot [°C]
Simulated ΔH change [kJ/mol]
1. maximum
2. maximum
12
212
262
412
112
g)
ΔG [kJ/mol]
Temperature [K]
 2.9 kJ/mol/K
 5.9 kJ/mol/K
 7.9 kJ/mol/K
12.9 kJ/mol/K
17.9 kJ/mol/K
h)
Ttrs, cold [°C]
Simulated ΔCp change [kJ/mol/K]
1. minimum
2. minimum
2.9
7.9
12.9
17.9
5.9
i)
Ttrs, hot [°C]
Simulated ΔCp change [kJ/mol/K]
1. maximum
2. maximum
2.9
7.9
12.9
17.9
5.9
Figure S5: Simulation of stability curve´s changes by varying of parameters a) Ttrs, hot; d) ΔH; g) ΔCp and determined Ttrs, cold (b; e; h) and Ttrs hot ( c; f; i).

## Slide 6
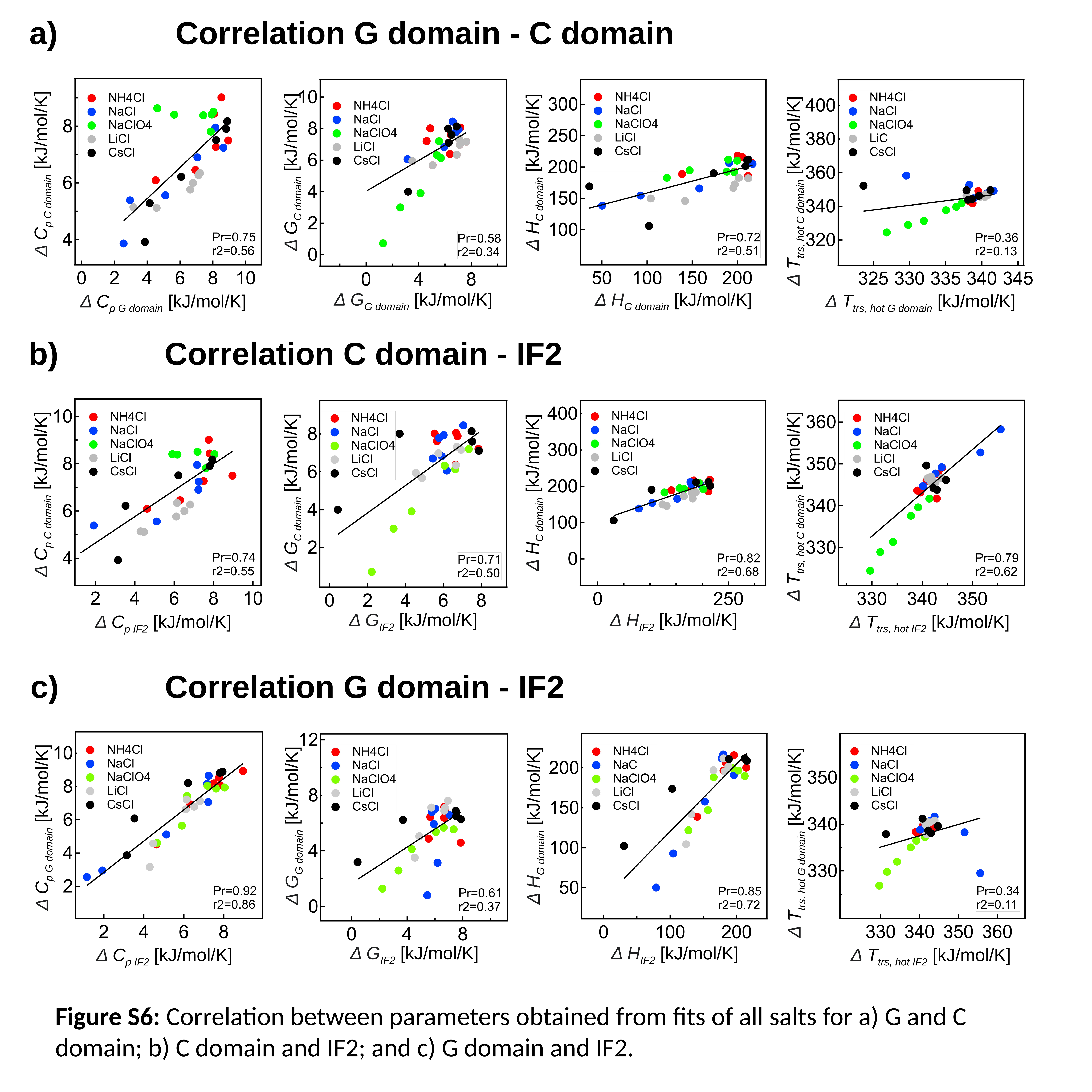

Correlation G domain - C domain
a)
Δ GC domain [kJ/mol/K]
Δ HC domain [kJ/mol/K]
Δ Cp C domain [kJ/mol/K]
Δ Ttrs, hot C domain [kJ/mol/K]
Δ GG domain [kJ/mol/K]
Δ HG domain [kJ/mol/K]
Δ Ttrs, hot G domain [kJ/mol/K]
Δ Cp G domain [kJ/mol/K]
Correlation C domain - IF2
b)
Δ GC domain [kJ/mol/K]
Δ HC domain [kJ/mol/K]
Δ Cp C domain [kJ/mol/K]
Δ Ttrs, hot C domain [kJ/mol/K]
Δ GIF2 [kJ/mol/K]
Δ HIF2 [kJ/mol/K]
Δ Ttrs, hot IF2 [kJ/mol/K]
Δ Cp IF2 [kJ/mol/K]
Correlation G domain - IF2
c)
Δ GG domain [kJ/mol/K]
Δ HG domain [kJ/mol/K]
Δ Cp G domain [kJ/mol/K]
Δ Ttrs, hot G domain [kJ/mol/K]
Δ GIF2 [kJ/mol/K]
Δ HIF2 [kJ/mol/K]
Δ Ttrs, hot IF2 [kJ/mol/K]
Δ Cp IF2 [kJ/mol/K]
Figure S6: Correlation between parameters obtained from fits of all salts for a) G and C domain; b) C domain and IF2; and c) G domain and IF2.

## Slide 7
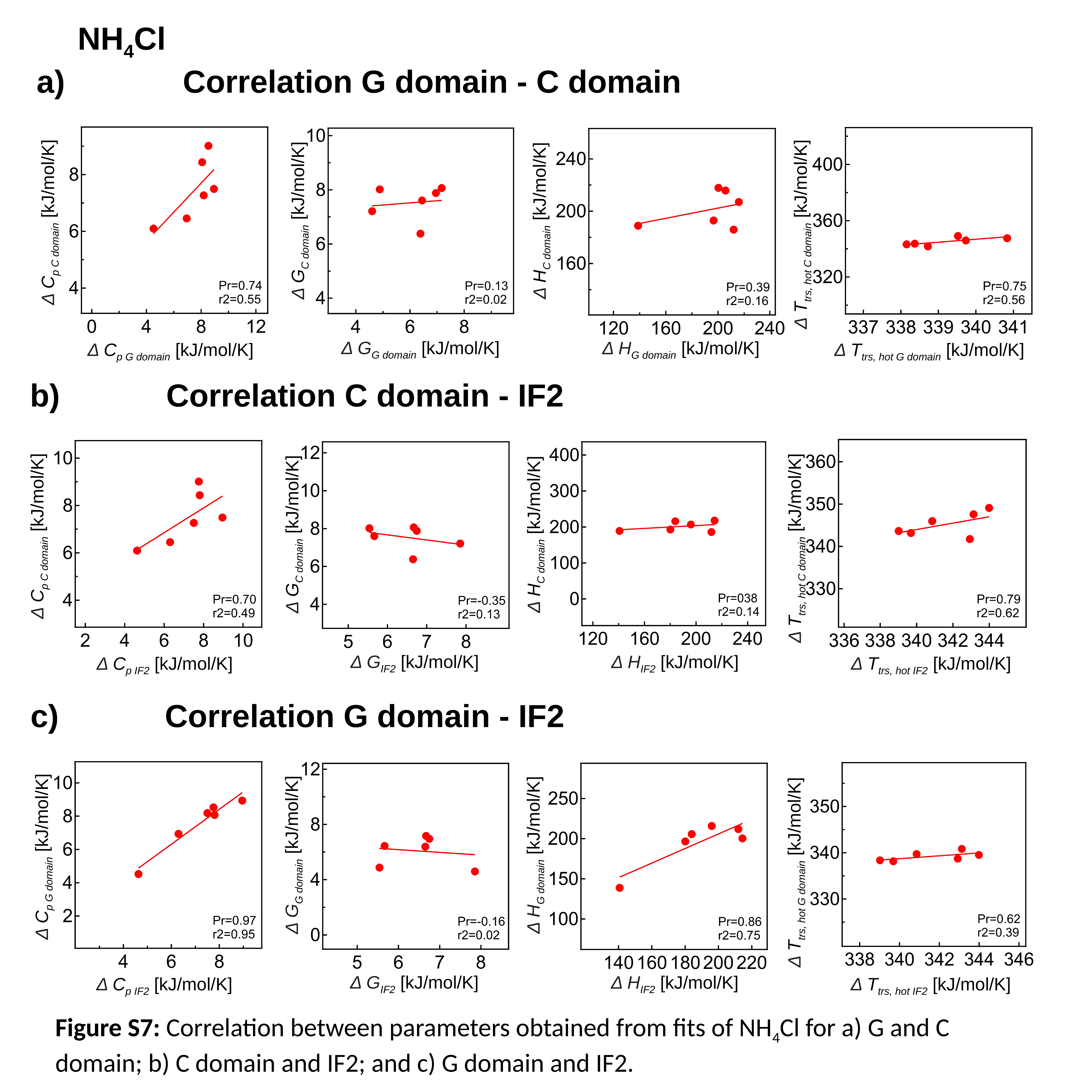

NH4Cl
Correlation G domain - C domain
a)
Δ GC domain [kJ/mol/K]
Δ HC domain [kJ/mol/K]
Δ Cp C domain [kJ/mol/K]
Δ Ttrs, hot C domain [kJ/mol/K]
Δ GG domain [kJ/mol/K]
Δ HG domain [kJ/mol/K]
Δ Ttrs, hot G domain [kJ/mol/K]
Δ Cp G domain [kJ/mol/K]
Correlation C domain - IF2
b)
Δ GC domain [kJ/mol/K]
Δ HC domain [kJ/mol/K]
Δ Cp C domain [kJ/mol/K]
Δ Ttrs, hot C domain [kJ/mol/K]
Δ GIF2 [kJ/mol/K]
Δ HIF2 [kJ/mol/K]
Δ Ttrs, hot IF2 [kJ/mol/K]
Δ Cp IF2 [kJ/mol/K]
Correlation G domain - IF2
c)
Δ GG domain [kJ/mol/K]
Δ HG domain [kJ/mol/K]
Δ Cp G domain [kJ/mol/K]
Δ Ttrs, hot G domain [kJ/mol/K]
Δ GIF2 [kJ/mol/K]
Δ HIF2 [kJ/mol/K]
Δ Ttrs, hot IF2 [kJ/mol/K]
Δ Cp IF2 [kJ/mol/K]
Figure S7: Correlation between parameters obtained from fits of NH4Cl for a) G and C domain; b) C domain and IF2; and c) G domain and IF2.

## Slide 8
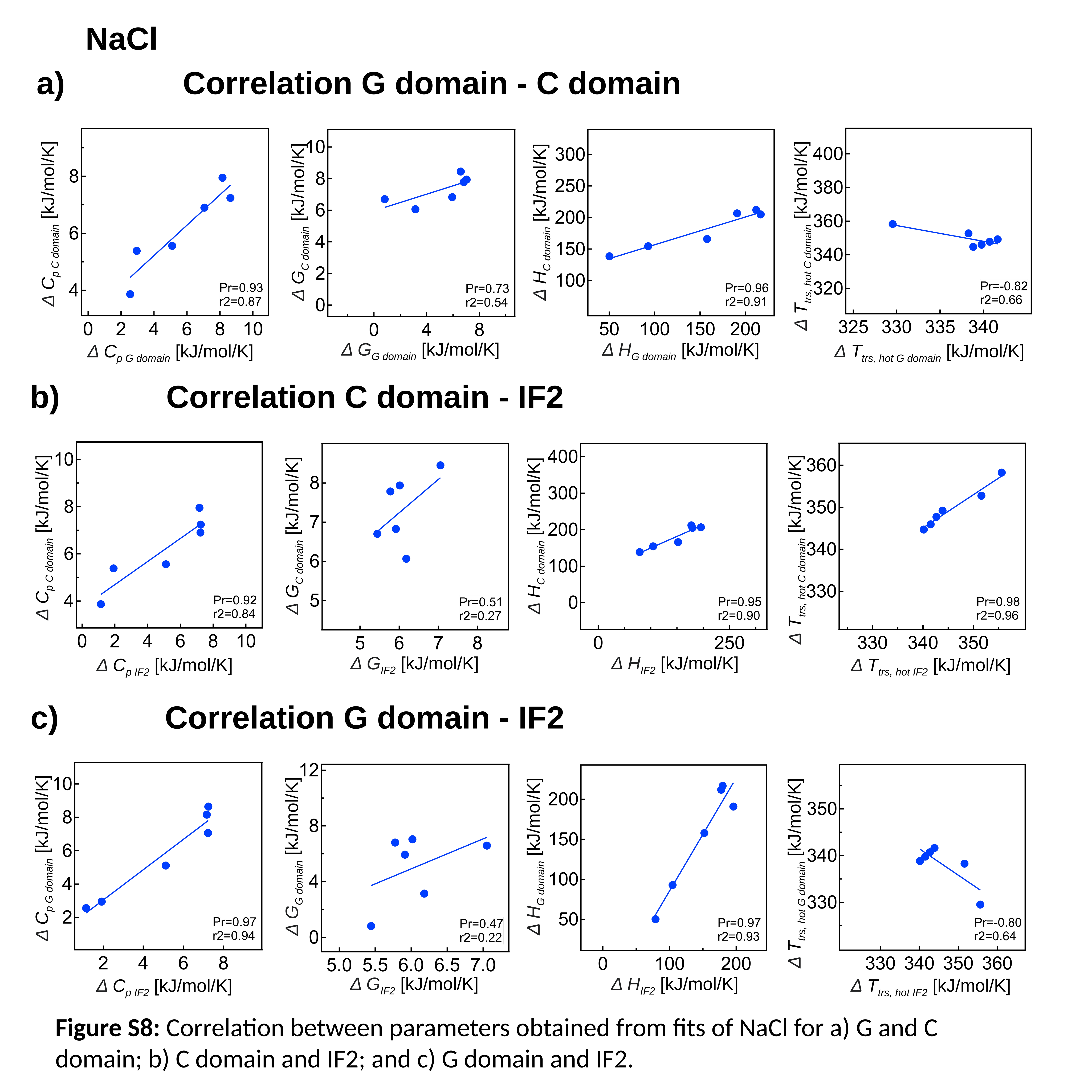

NaCl
Correlation G domain - C domain
a)
Δ GC domain [kJ/mol/K]
Δ HC domain [kJ/mol/K]
Δ Cp C domain [kJ/mol/K]
Δ Ttrs, hot C domain [kJ/mol/K]
Δ GG domain [kJ/mol/K]
Δ HG domain [kJ/mol/K]
Δ Ttrs, hot G domain [kJ/mol/K]
Δ Cp G domain [kJ/mol/K]
Correlation C domain - IF2
b)
Δ GC domain [kJ/mol/K]
Δ HC domain [kJ/mol/K]
Δ Cp C domain [kJ/mol/K]
Δ Ttrs, hot C domain [kJ/mol/K]
Δ GIF2 [kJ/mol/K]
Δ HIF2 [kJ/mol/K]
Δ Ttrs, hot IF2 [kJ/mol/K]
Δ Cp IF2 [kJ/mol/K]
Correlation G domain - IF2
c)
Δ GG domain [kJ/mol/K]
Δ HG domain [kJ/mol/K]
Δ Cp G domain [kJ/mol/K]
Δ Ttrs, hot G domain [kJ/mol/K]
Δ GIF2 [kJ/mol/K]
Δ HIF2 [kJ/mol/K]
Δ Ttrs, hot IF2 [kJ/mol/K]
Δ Cp IF2 [kJ/mol/K]
Figure S8: Correlation between parameters obtained from fits of NaCl for a) G and C domain; b) C domain and IF2; and c) G domain and IF2.

## Slide 9
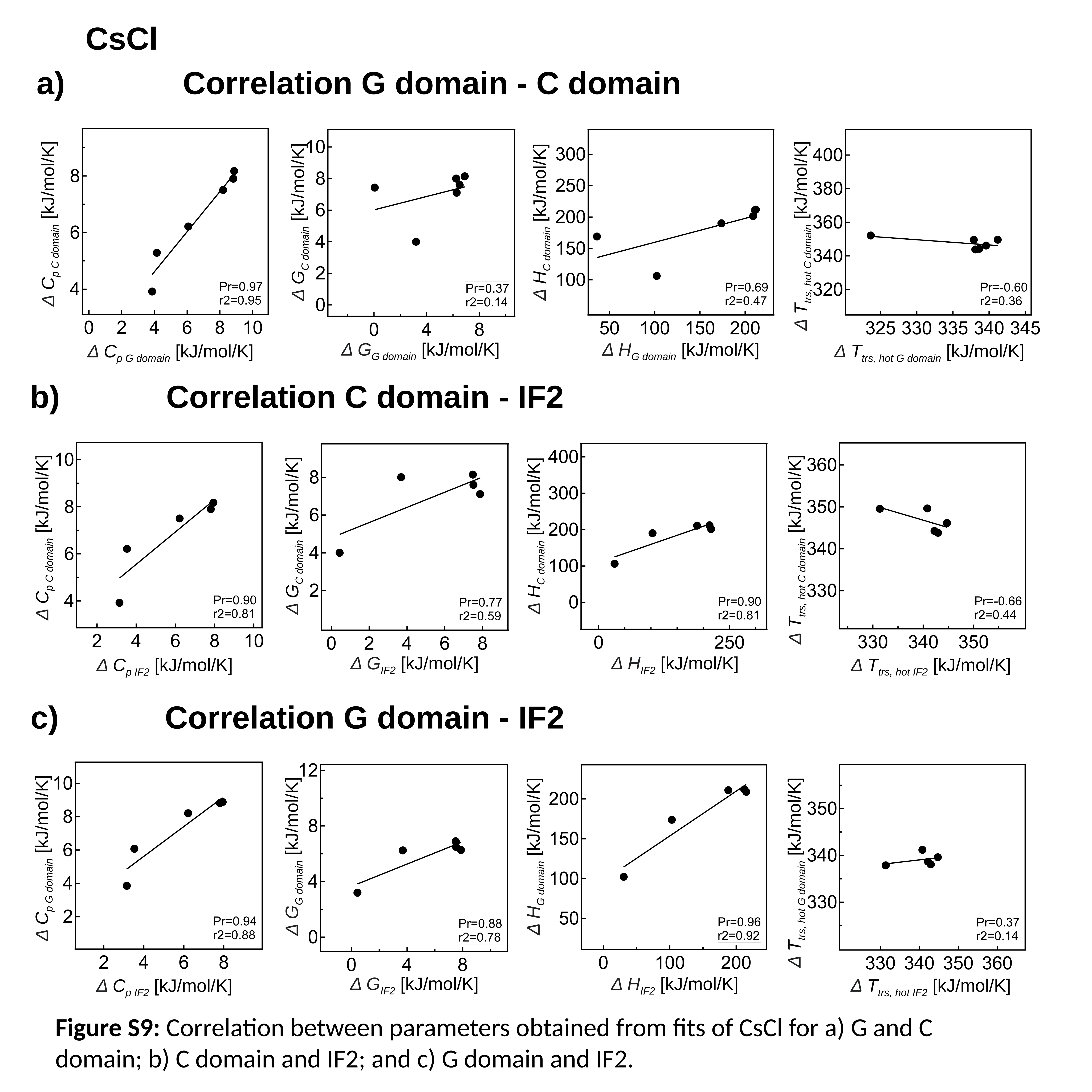

CsCl
Correlation G domain - C domain
a)
Δ GC domain [kJ/mol/K]
Δ HC domain [kJ/mol/K]
Δ Cp C domain [kJ/mol/K]
Δ Ttrs, hot C domain [kJ/mol/K]
Δ GG domain [kJ/mol/K]
Δ HG domain [kJ/mol/K]
Δ Ttrs, hot G domain [kJ/mol/K]
Δ Cp G domain [kJ/mol/K]
Correlation C domain - IF2
b)
Δ GC domain [kJ/mol/K]
Δ HC domain [kJ/mol/K]
Δ Cp C domain [kJ/mol/K]
Δ Ttrs, hot C domain [kJ/mol/K]
Δ GIF2 [kJ/mol/K]
Δ HIF2 [kJ/mol/K]
Δ Ttrs, hot IF2 [kJ/mol/K]
Δ Cp IF2 [kJ/mol/K]
Correlation G domain - IF2
c)
Δ GG domain [kJ/mol/K]
Δ HG domain [kJ/mol/K]
Δ Cp G domain [kJ/mol/K]
Δ Ttrs, hot G domain [kJ/mol/K]
Δ GIF2 [kJ/mol/K]
Δ HIF2 [kJ/mol/K]
Δ Ttrs, hot IF2 [kJ/mol/K]
Δ Cp IF2 [kJ/mol/K]
Figure S9: Correlation between parameters obtained from fits of CsCl for a) G and C domain; b) C domain and IF2; and c) G domain and IF2.

## Slide 10
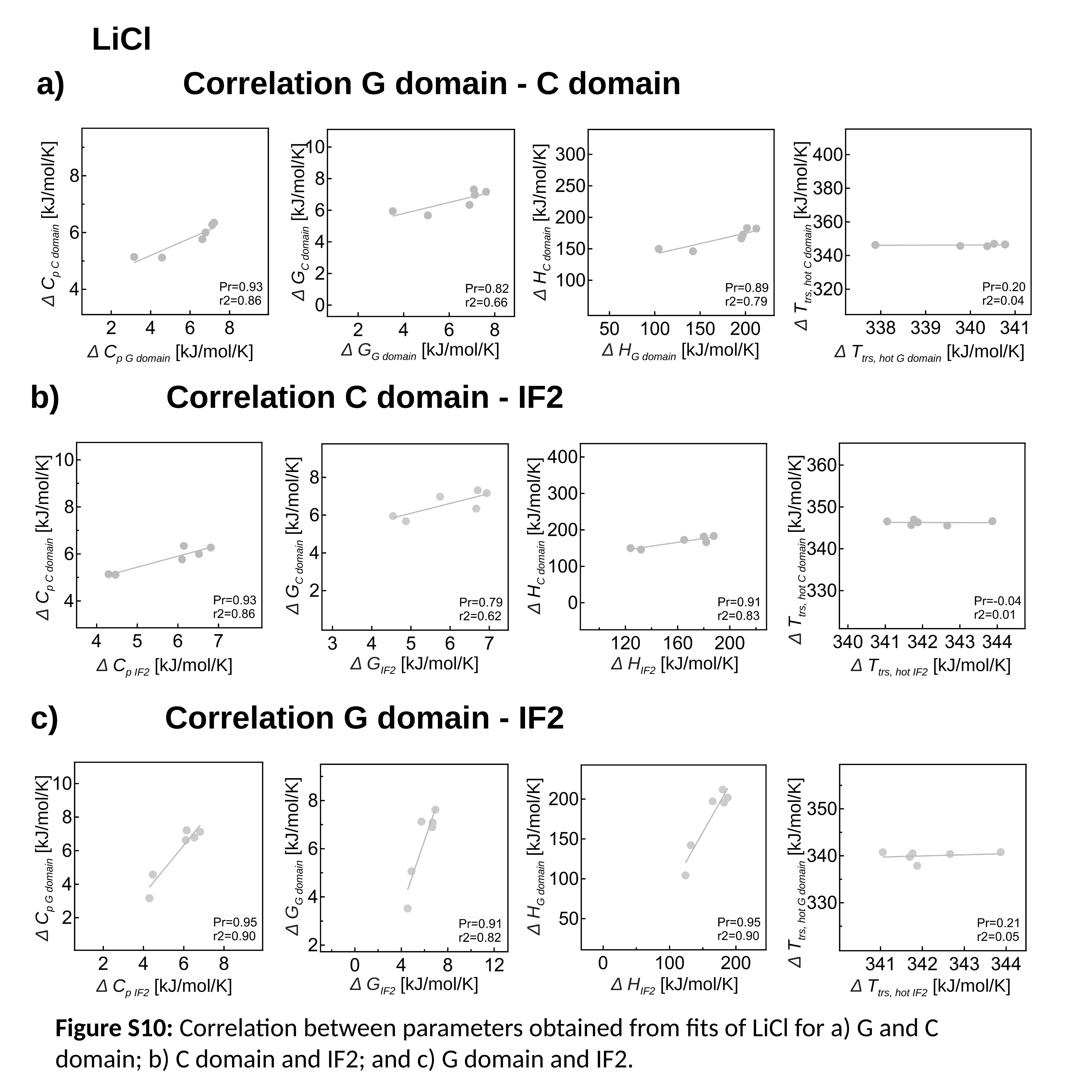

LiCl
Correlation G domain - C domain
a)
Δ GC domain [kJ/mol/K]
Δ HC domain [kJ/mol/K]
Δ Cp C domain [kJ/mol/K]
Δ Ttrs, hot C domain [kJ/mol/K]
Δ GG domain [kJ/mol/K]
Δ HG domain [kJ/mol/K]
Δ Ttrs, hot G domain [kJ/mol/K]
Δ Cp G domain [kJ/mol/K]
Correlation C domain - IF2
b)
Δ GC domain [kJ/mol/K]
Δ HC domain [kJ/mol/K]
Δ Cp C domain [kJ/mol/K]
Δ Ttrs, hot C domain [kJ/mol/K]
Δ GIF2 [kJ/mol/K]
Δ HIF2 [kJ/mol/K]
Δ Ttrs, hot IF2 [kJ/mol/K]
Δ Cp IF2 [kJ/mol/K]
Correlation G domain - IF2
c)
Δ GG domain [kJ/mol/K]
Δ HG domain [kJ/mol/K]
Δ Cp G domain [kJ/mol/K]
Δ Ttrs, hot G domain [kJ/mol/K]
Δ GIF2 [kJ/mol/K]
Δ HIF2 [kJ/mol/K]
Δ Ttrs, hot IF2 [kJ/mol/K]
Δ Cp IF2 [kJ/mol/K]
Figure S10: Correlation between parameters obtained from fits of LiCl for a) G and C domain; b) C domain and IF2; and c) G domain and IF2.

## Slide 11
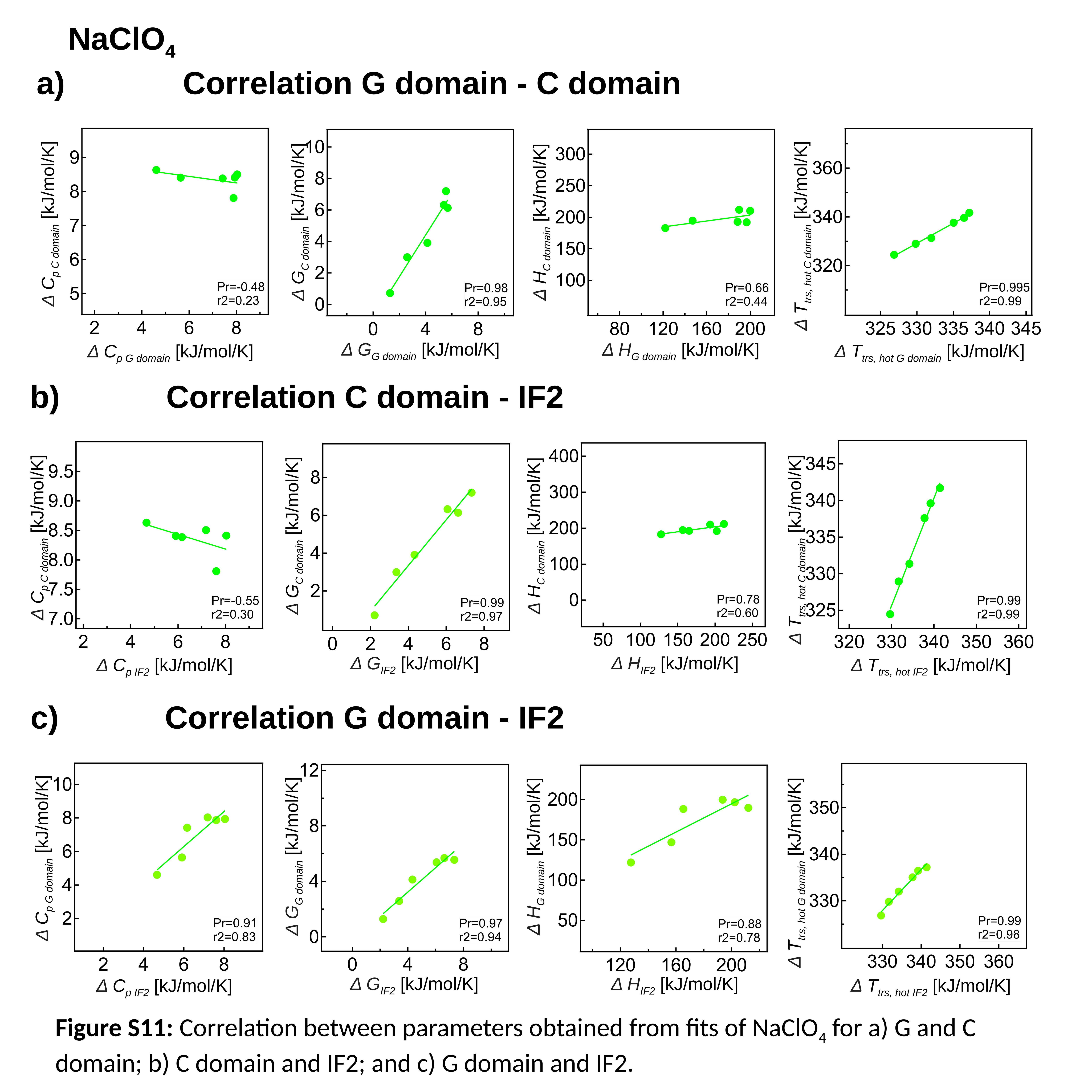

NaClO4
Correlation G domain - C domain
a)
Δ GC domain [kJ/mol/K]
Δ HC domain [kJ/mol/K]
Δ Cp C domain [kJ/mol/K]
Δ Ttrs, hot C domain [kJ/mol/K]
Δ GG domain [kJ/mol/K]
Δ HG domain [kJ/mol/K]
Δ Ttrs, hot G domain [kJ/mol/K]
Δ Cp G domain [kJ/mol/K]
Correlation C domain - IF2
b)
Δ GC domain [kJ/mol/K]
Δ HC domain [kJ/mol/K]
Δ Cp C domain [kJ/mol/K]
Δ Ttrs, hot C domain [kJ/mol/K]
Δ GIF2 [kJ/mol/K]
Δ HIF2 [kJ/mol/K]
Δ Ttrs, hot IF2 [kJ/mol/K]
Δ Cp IF2 [kJ/mol/K]
Correlation G domain - IF2
c)
Δ GG domain [kJ/mol/K]
Δ HG domain [kJ/mol/K]
Δ Cp G domain [kJ/mol/K]
Δ Ttrs, hot G domain [kJ/mol/K]
Δ GIF2 [kJ/mol/K]
Δ HIF2 [kJ/mol/K]
Δ Ttrs, hot IF2 [kJ/mol/K]
Δ Cp IF2 [kJ/mol/K]
Figure S11: Correlation between parameters obtained from fits of NaClO4 for a) G and C domain; b) C domain and IF2; and c) G domain and IF2.

## Slide 12
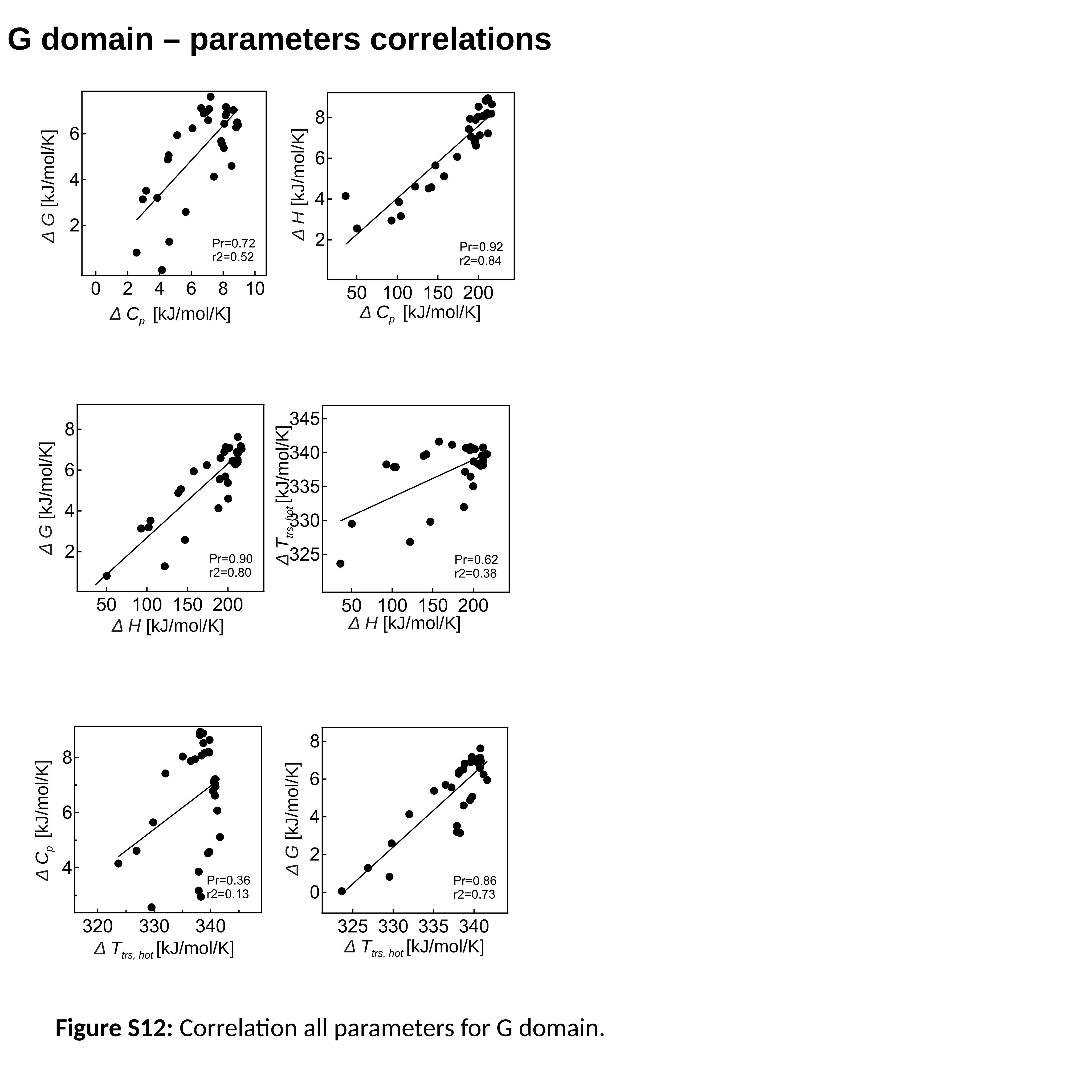

G domain – parameters correlations
Δ H [kJ/mol/K]
Δ G [kJ/mol/K]
Δ Cp [kJ/mol/K]
Δ Cp [kJ/mol/K]
Δ Ttrs, hot [kJ/mol/K]
Δ G [kJ/mol/K]
Δ H [kJ/mol/K]
Δ H [kJ/mol/K]
Δ G [kJ/mol/K]
Δ Cp [kJ/mol/K]
Δ Ttrs, hot [kJ/mol/K]
Δ Ttrs, hot [kJ/mol/K]
Figure S12: Correlation all parameters for G domain.

## Slide 13
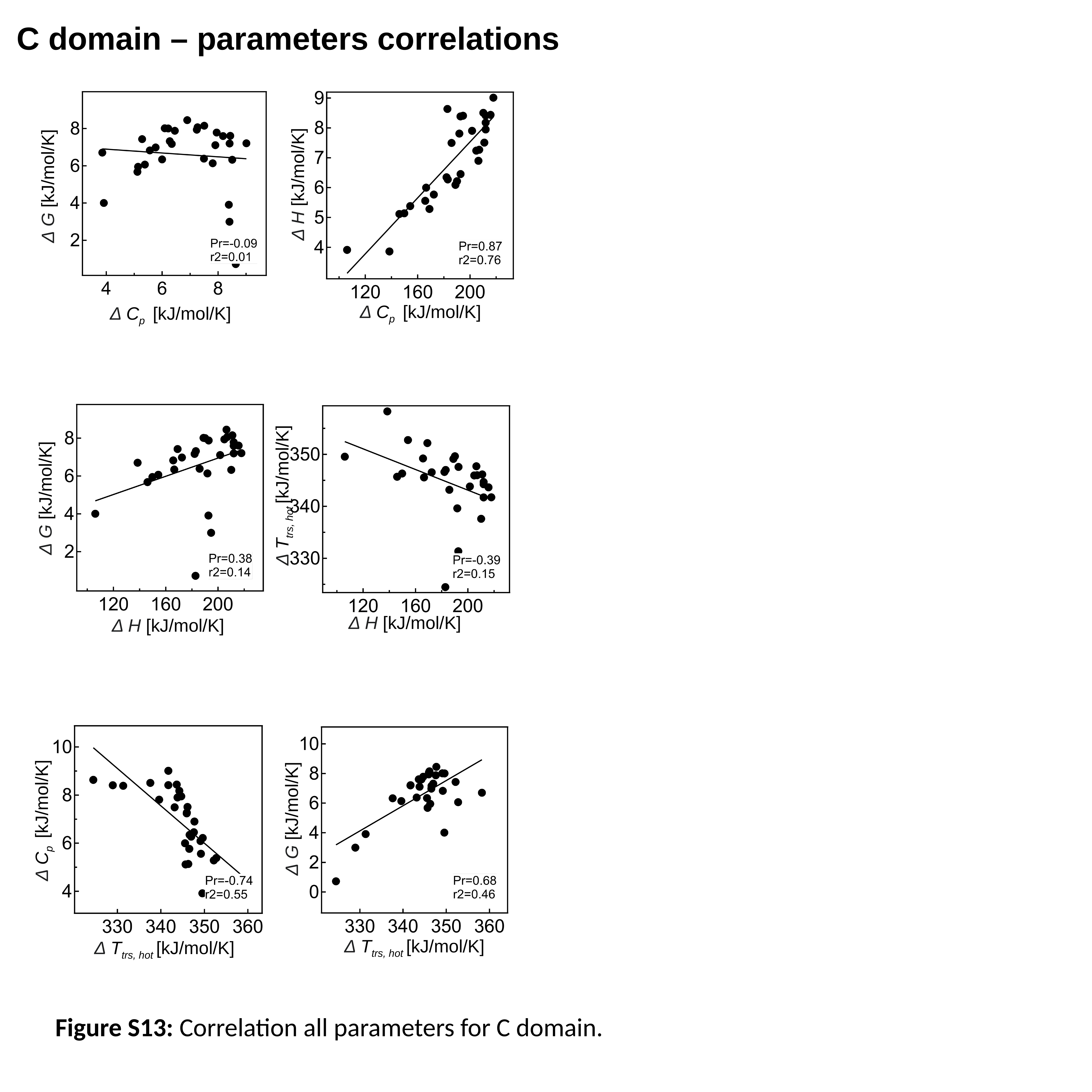

C domain – parameters correlations
Δ H [kJ/mol/K]
Δ G [kJ/mol/K]
Δ Cp [kJ/mol/K]
Δ Cp [kJ/mol/K]
Δ Ttrs, hot [kJ/mol/K]
Δ G [kJ/mol/K]
Δ H [kJ/mol/K]
Δ H [kJ/mol/K]
Δ G [kJ/mol/K]
Δ Cp [kJ/mol/K]
Δ Ttrs, hot [kJ/mol/K]
Δ Ttrs, hot [kJ/mol/K]
Figure S13: Correlation all parameters for C domain.

## Slide 14
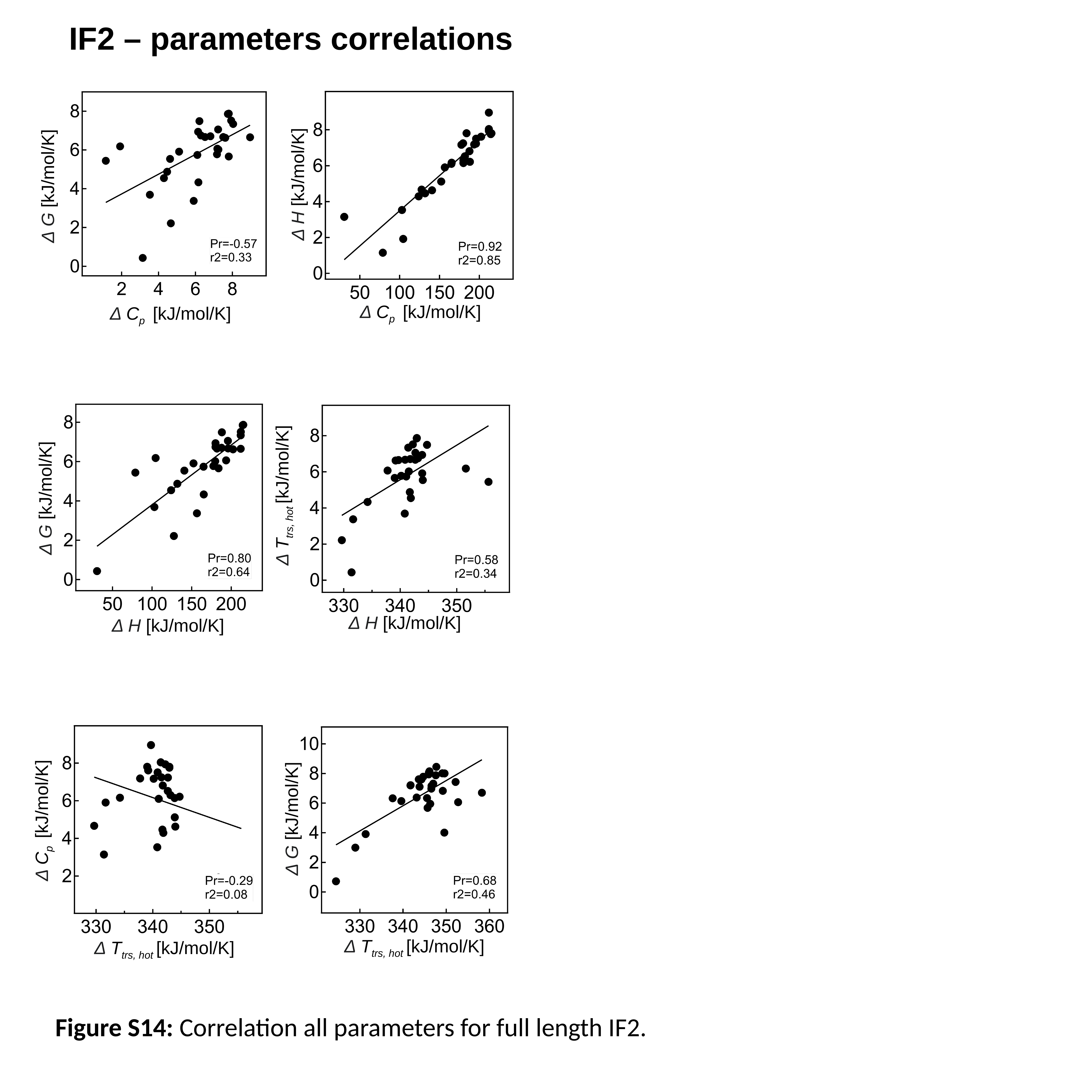

IF2 – parameters correlations
Δ H [kJ/mol/K]
Δ G [kJ/mol/K]
Δ Cp [kJ/mol/K]
Δ Cp [kJ/mol/K]
Δ Ttrs, hot [kJ/mol/K]
Δ G [kJ/mol/K]
Δ H [kJ/mol/K]
Δ H [kJ/mol/K]
Δ G [kJ/mol/K]
Δ Cp [kJ/mol/K]
Δ Ttrs, hot [kJ/mol/K]
Δ Ttrs, hot [kJ/mol/K]
Figure S14: Correlation all parameters for full length IF2.

## Slide 15
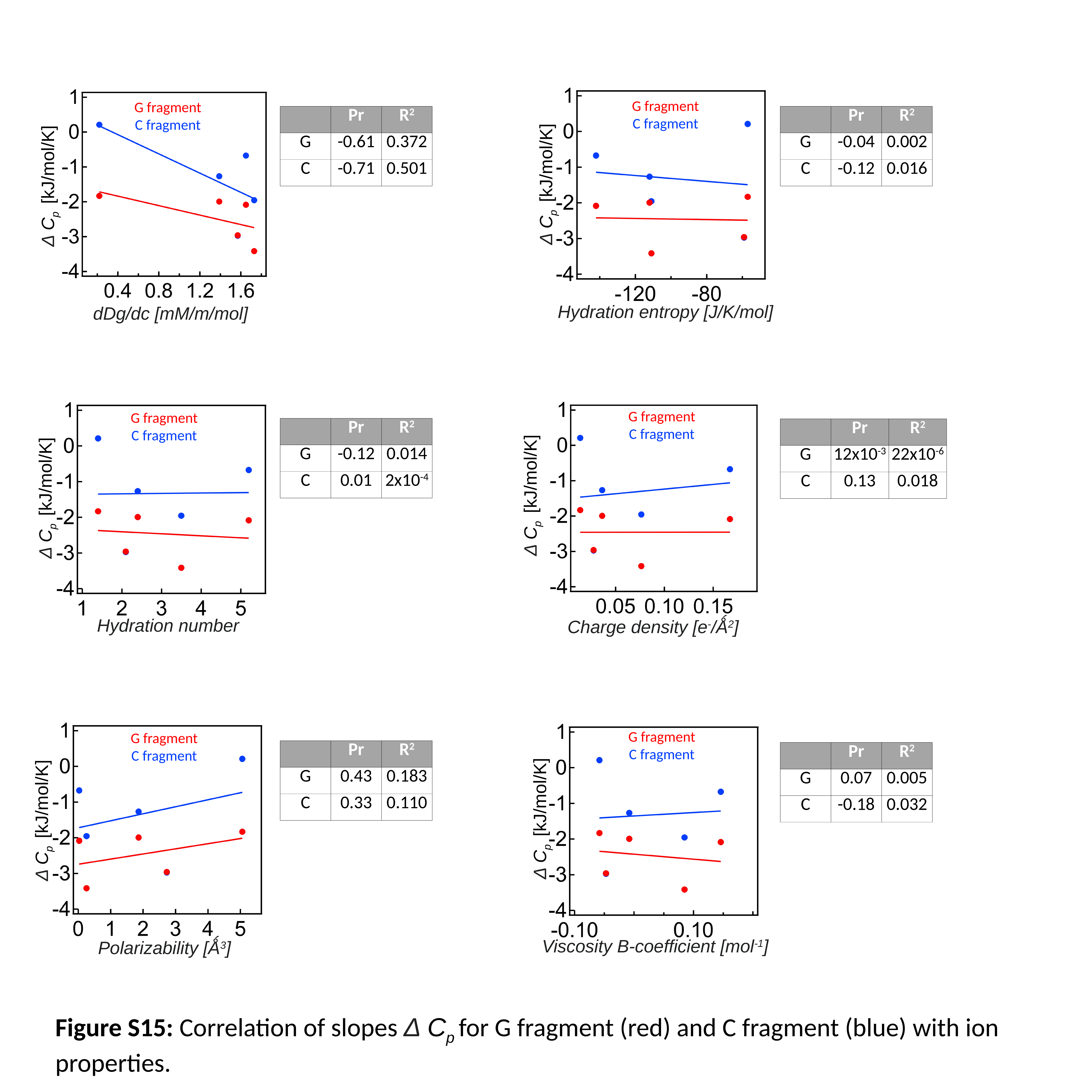

G fragment
C fragment
G fragment
C fragment
| | Pr | R2 |
| --- | --- | --- |
| G | -0.61 | 0.372 |
| C | -0.71 | 0.501 |
| | Pr | R2 |
| --- | --- | --- |
| G | -0.04 | 0.002 |
| C | -0.12 | 0.016 |
Δ Cp [kJ/mol/K]
Δ Cp [kJ/mol/K]
Hydration entropy [J/K/mol]
dDg/dc [mM/m/mol]
G fragment
C fragment
G fragment
C fragment
| | Pr | R2 |
| --- | --- | --- |
| G | -0.12 | 0.014 |
| C | 0.01 | 2x10^-4^ |
| | Pr | R2 |
| --- | --- | --- |
| G | 12x10-3 | 22x10-6 |
| C | 0.13 | 0.018 |
Δ Cp [kJ/mol/K]
Δ Cp [kJ/mol/K]
Hydration number
Charge density [e-/Ǻ2]
G fragment
C fragment
G fragment
C fragment
| | Pr | R2 |
| --- | --- | --- |
| G | 0.43 | 0.183 |
| C | 0.33 | 0.110 |
| | Pr | R2 |
| --- | --- | --- |
| G | 0.07 | 0.005 |
| C | -0.18 | 0.032 |
Δ Cp [kJ/mol/K]
Δ Cp [kJ/mol/K]
Viscosity B-coefficient [mol-1]
Polarizability [Ǻ3]
Figure S15: Correlation of slopes Δ Cp for G fragment (red) and C fragment (blue) with ion properties.

## Slide 16
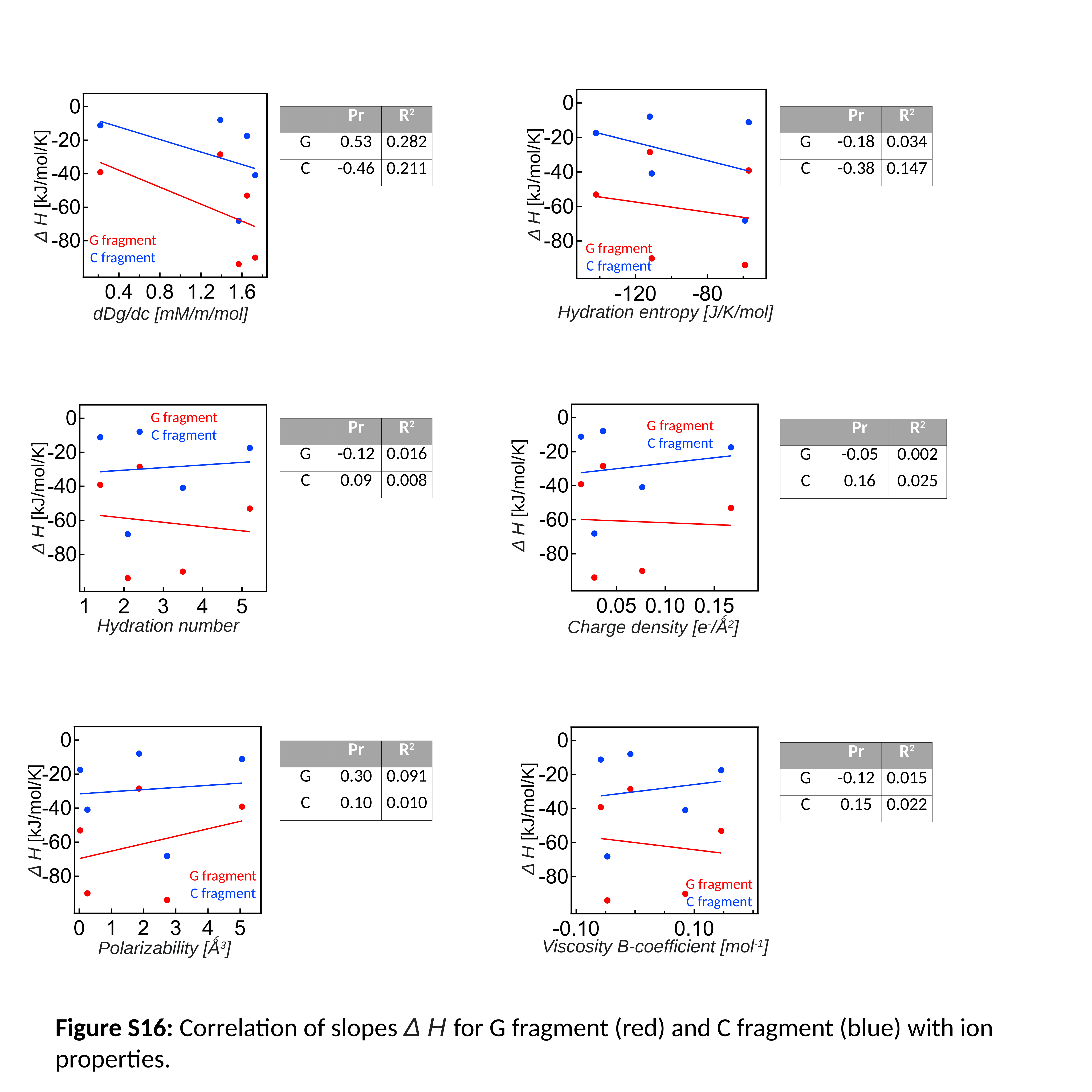

| | Pr | R2 |
| --- | --- | --- |
| G | 0.53 | 0.282 |
| C | -0.46 | 0.211 |
| | Pr | R2 |
| --- | --- | --- |
| G | -0.18 | 0.034 |
| C | -0.38 | 0.147 |
Δ H [kJ/mol/K]
Δ H [kJ/mol/K]
G fragment
C fragment
G fragment
C fragment
Hydration entropy [J/K/mol]
dDg/dc [mM/m/mol]
G fragment
C fragment
G fragment
C fragment
| | Pr | R2 |
| --- | --- | --- |
| G | -0.12 | 0.016 |
| C | 0.09 | 0.008 |
| | Pr | R2 |
| --- | --- | --- |
| G | -0.05 | 0.002 |
| C | 0.16 | 0.025 |
Δ H [kJ/mol/K]
Δ H [kJ/mol/K]
Hydration number
Charge density [e-/Ǻ2]
| | Pr | R2 |
| --- | --- | --- |
| G | 0.30 | 0.091 |
| C | 0.10 | 0.010 |
| | Pr | R2 |
| --- | --- | --- |
| G | -0.12 | 0.015 |
| C | 0.15 | 0.022 |
Δ H [kJ/mol/K]
Δ H [kJ/mol/K]
G fragment
C fragment
G fragment
C fragment
Viscosity B-coefficient [mol-1]
Polarizability [Ǻ3]
Figure S16: Correlation of slopes Δ H for G fragment (red) and C fragment (blue) with ion properties.

## Slide 17
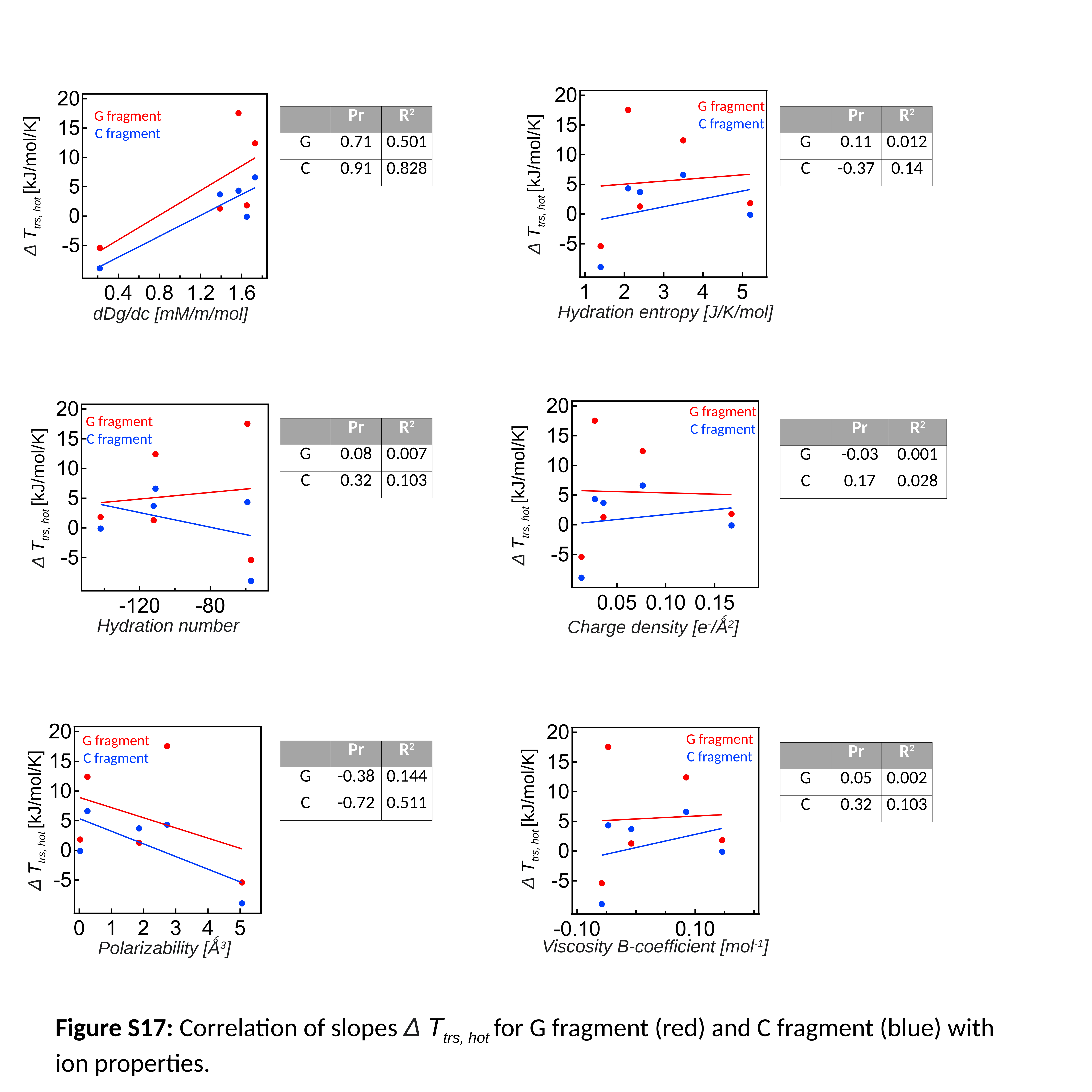

G fragment
C fragment
G fragment
C fragment
| | Pr | R2 |
| --- | --- | --- |
| G | 0.71 | 0.501 |
| C | 0.91 | 0.828 |
| | Pr | R2 |
| --- | --- | --- |
| G | 0.11 | 0.012 |
| C | -0.37 | 0.14 |
Δ Ttrs, hot [kJ/mol/K]
Δ Ttrs, hot [kJ/mol/K]
Hydration entropy [J/K/mol]
dDg/dc [mM/m/mol]
G fragment
C fragment
G fragment
C fragment
| | Pr | R2 |
| --- | --- | --- |
| G | 0.08 | 0.007 |
| C | 0.32 | 0.103 |
| | Pr | R2 |
| --- | --- | --- |
| G | -0.03 | 0.001 |
| C | 0.17 | 0.028 |
Δ Ttrs, hot [kJ/mol/K]
Δ Ttrs, hot [kJ/mol/K]
Hydration number
Charge density [e-/Ǻ2]
G fragment
C fragment
G fragment
C fragment
| | Pr | R2 |
| --- | --- | --- |
| G | -0.38 | 0.144 |
| C | -0.72 | 0.511 |
| | Pr | R2 |
| --- | --- | --- |
| G | 0.05 | 0.002 |
| C | 0.32 | 0.103 |
Δ Ttrs, hot [kJ/mol/K]
Δ Ttrs, hot [kJ/mol/K]
Viscosity B-coefficient [mol-1]
Polarizability [Ǻ3]
Figure S17: Correlation of slopes Δ Ttrs, hot for G fragment (red) and C fragment (blue) with ion properties.

## Slide 18
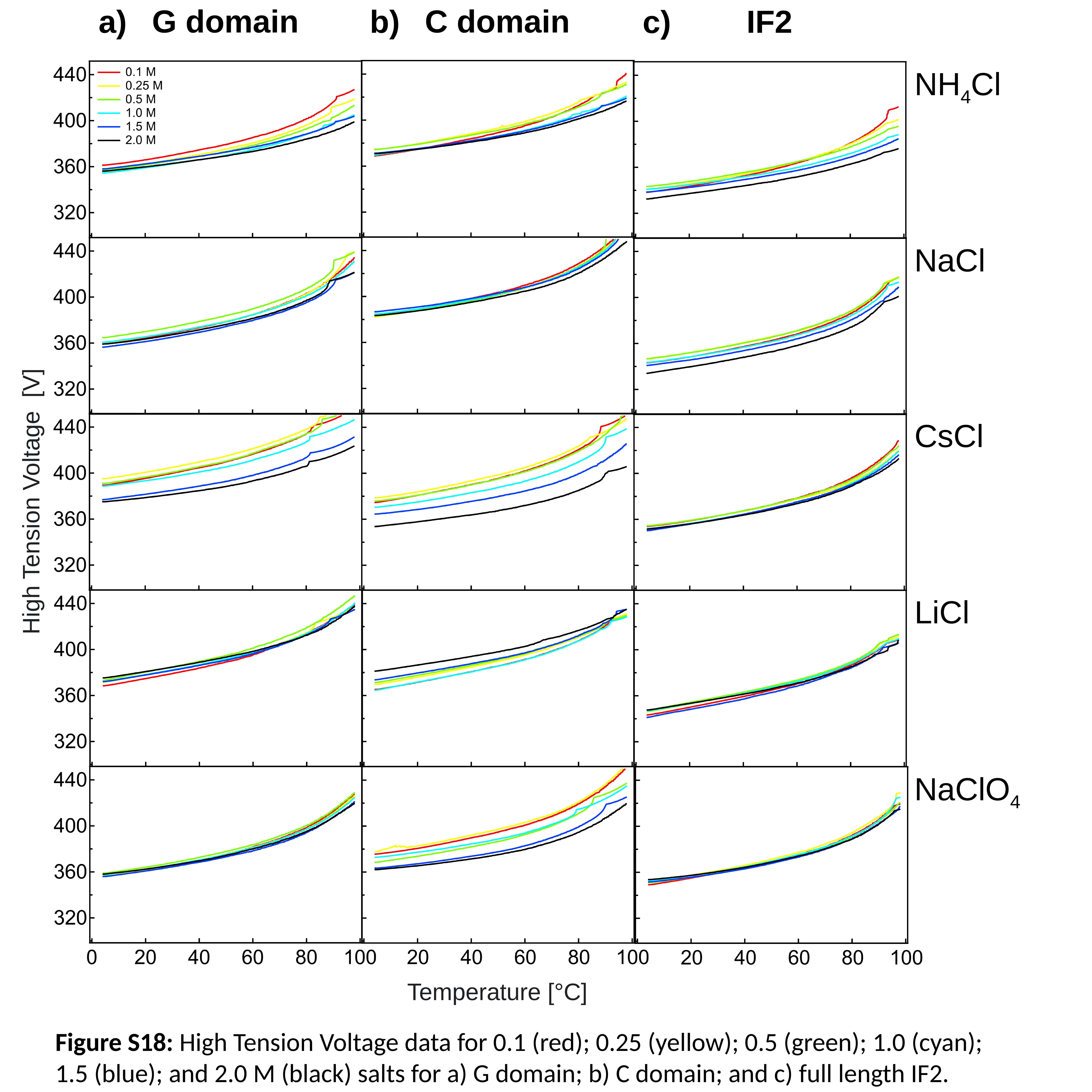

a)
b)
IF2
G domain
C domain
c)
NH4Cl
NaCl
CsCl
High Tension Voltage [V]
LiCl
NaClO4
Temperature [°C]
Figure S18: High Tension Voltage data for 0.1 (red); 0.25 (yellow); 0.5 (green); 1.0 (cyan); 1.5 (blue); and 2.0 M (black) salts for a) G domain; b) C domain; and c) full length IF2.

## Slide 19
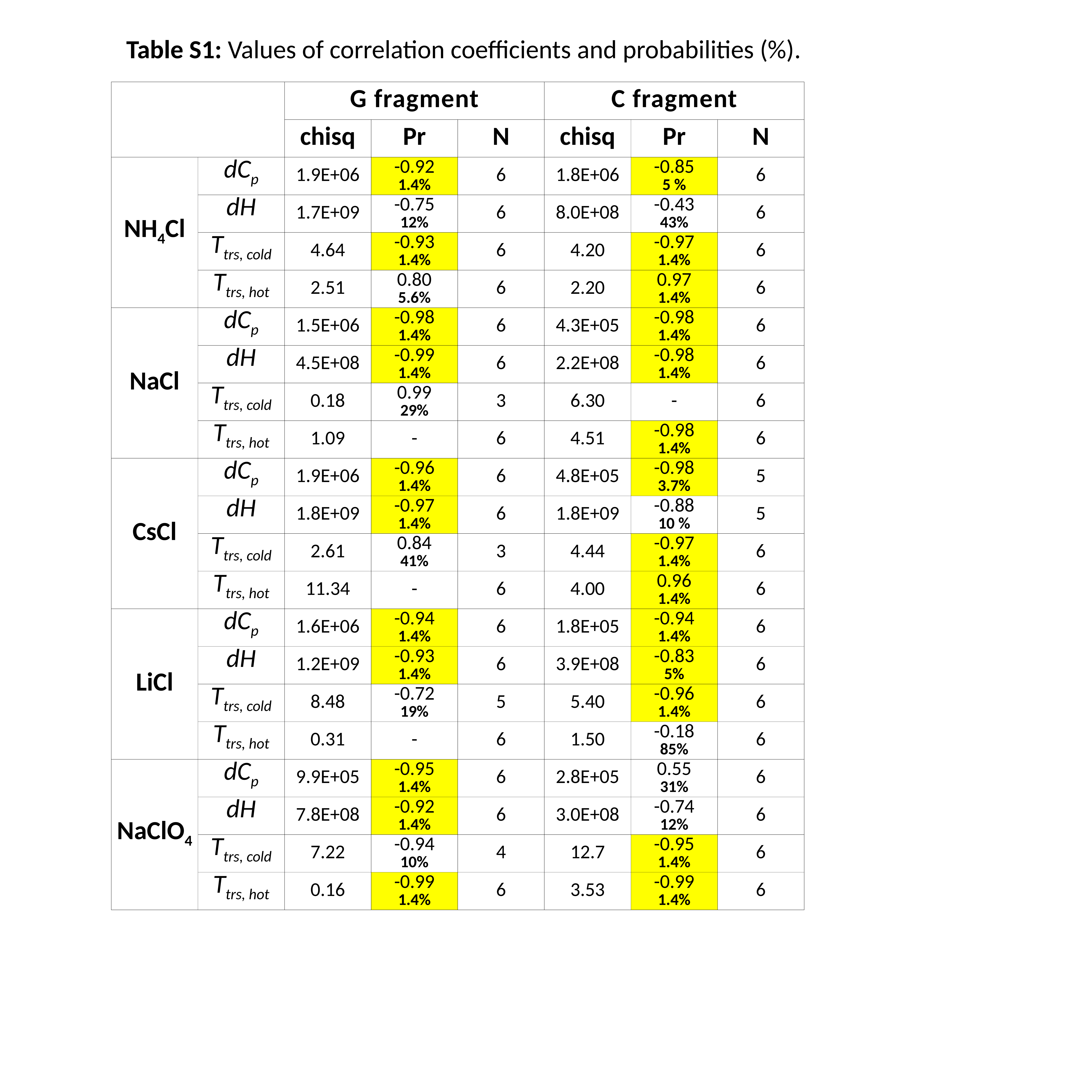

Table S1: Values of correlation coefficients and probabilities (%).
| | | G fragment | | | C fragment | | |
| --- | --- | --- | --- | --- | --- | --- | --- |
| | | chisq | Pr | N | chisq | Pr | N |
| NH4Cl | dCp | 1.9E+06 | -0.92 1.4% | 6 | 1.8E+06 | -0.85 5 % | 6 |
| | dH | 1.7E+09 | -0.75 12% | 6 | 8.0E+08 | -0.43 43% | 6 |
| | Ttrs, cold | 4.64 | -0.93 1.4% | 6 | 4.20 | -0.97 1.4% | 6 |
| | Ttrs, hot | 2.51 | 0.80 5.6% | 6 | 2.20 | 0.97 1.4% | 6 |
| NaCl | dCp | 1.5E+06 | -0.98 1.4% | 6 | 4.3E+05 | -0.98 1.4% | 6 |
| | dH | 4.5E+08 | -0.99 1.4% | 6 | 2.2E+08 | -0.98 1.4% | 6 |
| | Ttrs, cold | 0.18 | 0.99 29% | 3 | 6.30 | - | 6 |
| | Ttrs, hot | 1.09 | - | 6 | 4.51 | -0.98 1.4% | 6 |
| CsCl | dCp | 1.9E+06 | -0.96 1.4% | 6 | 4.8E+05 | -0.98 3.7% | 5 |
| | dH | 1.8E+09 | -0.97 1.4% | 6 | 1.8E+09 | -0.88 10 % | 5 |
| | Ttrs, cold | 2.61 | 0.84 41% | 3 | 4.44 | -0.97 1.4% | 6 |
| | Ttrs, hot | 11.34 | - | 6 | 4.00 | 0.96 1.4% | 6 |
| LiCl | dCp | 1.6E+06 | -0.94 1.4% | 6 | 1.8E+05 | -0.94 1.4% | 6 |
| | dH | 1.2E+09 | -0.93 1.4% | 6 | 3.9E+08 | -0.83 5% | 6 |
| | Ttrs, cold | 8.48 | -0.72 19% | 5 | 5.40 | -0.96 1.4% | 6 |
| | Ttrs, hot | 0.31 | - | 6 | 1.50 | -0.18 85% | 6 |
| NaClO4 | dCp | 9.9E+05 | -0.95 1.4% | 6 | 2.8E+05 | 0.55 31% | 6 |
| | dH | 7.8E+08 | -0.92 1.4% | 6 | 3.0E+08 | -0.74 12% | 6 |
| | Ttrs, cold | 7.22 | -0.94 10% | 4 | 12.7 | -0.95 1.4% | 6 |
| | Ttrs, hot | 0.16 | -0.99 1.4% | 6 | 3.53 | -0.99 1.4% | 6 |
